# Association between Polymorphisms in IL-16 Genes and Coronary Heart Disease risk

**DOI:** 10.12669/pjms.294.3650

**Published:** 2013

**Authors:** Tan Hai-feng, Wang Wei, Yang Yuan-yuan, Zhao Jun, Gong Su-ping, Li Hui-ming

**Affiliations:** 1 Tan Hai-feng, Physical Examination Center, Second People’s Hospital of Ji’nan, Ji’nan, China.; 2 Wang Wei,Department of Community, Second People’s Hospital of Ji’nan, Ji’nan, China.; 3 Yang Yuan-yuan, Physical Examination Center, Second People’s Hospital of Ji’nan, Ji’nan, China.; 4 Zhao Jun, Physical Examination Center, Second People’s Hospital of Ji’nan, Ji’nan, China.; 5 Gong Su-ping, Physical Examination Center, Second People’s Hospital of Ji’nan, Ji’nan, China.; 6 Li Hui-ming, Department of Community, Second People’s Hospital of Ji’nan, Ji’nan, China.

**Keywords:** Coronary artery disease, IL-16, Polymorphism, Predictor

## Abstract

***Objective:*** We aimed to investigate the role of polymorphisms in IL-16 genes on the susceptibility of Coronary Artery Disease (CAD).

***Methods:*** A total of 260 CAD cases and 281 health controls were collected between January 2008 and November 2011. Genotyping of IL-16 rs8034928, rs3848180, rs1131445, rs4778889 and rs11556218 was conducted by polymerase chain reaction (PCR) and matrix-assisted laser desorption/ionization time-of-flight (MALDI-TOF) mass spectrometry technologies.

***Results:*** The frequencies of rs8034928 C allele and rs3848180 G allele in the CAD cases in CAD group were significantly higher than in controls. Compared with rs8034928 T/T genotype, a significant higher risk of CAD was found in C/C genotype (OR=1.87, 95%CI=1.17-3.03), and variant of rs8034928 showed a significant increased risk of CAD in dominant (OR=1.48, 95%CI=1.04-2.10) and recessive model (OR=1.70, 95%CI=1.10-2.67). The rs3848180 G/G was found to be associated with risk of CAD(OR=1.79, 95%CI=1.16-2.75), and G allele carries had a significant risk of CAD (OR=1.47, 95%CI=1.02-2.13).

***Conclusions:*** Our study indicated that rs8034928 and rs11556218 polymorphisms are associated with CAD risk in a Chinese population, and IL-16 gene polymorphisms may be used as a predictor to the susceptibility of CAD.

## INTRODUCTION

Coronary artery disease (CAD) is a serious national health problem both in developed and developing country.^1^ CAD is considered to be caused by various factors, such as inflammation, smoking, hypertension, diabetes and genetic factors.^2^^,^^3^ It is reported that the underlying pathological process of CAD is atherosclerosis, and atherosclerosis is a chronic inflammation induced by the deposit of oxidized lipids on the inner layer of the arterial wall.^3^ Previous studies have shown that many inflammation related genes, such as interlukin-16 (IL-16) and interlukin-6 (IL-6), are susceptible to the risk of CAD.^4^

Interleukin-16 (IL-16) is a proinflammatory and immunoregulatory cytokine, and is reported to be related with the inflammation and immunity.^3^ IL-16 is a T-cell chemoattractant factor, and located on chromosome 15q26.3 and translated into a 631-amino acid precursor protein.^5^ Several recent studies have shown the variants of IL-16 gene are associated with various cancers risk and autoimmune diseases, such as rs11556218 and rs3848180.^6^^-^^8^ Previous study has indicated that the levels of IL-6 and its final product, C reactive protein, have been associated with increased CAD risk.^9^ The polymorphism of this gene could increase the concentration of IL-6 in plasma and CRP levels, and thus increase the inflammation process and blood pressure. However, the results are inconsistent.^3^^,^^9^^,^^10^ Two previous studies reported the negative association between polymorphisms of IL-6 and risk of CAD. ^9^^,^^10^

Therefore, the present study was aimed to investigate the role of polymorphism in IL-16 (rs8034928, rs3848180, rs1131445, rs4778889 and rs11556218) on the susceptibility of CAD in a case-control study.

## METHODS

***Patients*** *: *This case-control study enrolled 260 CAD cases and 281 health controls. 308 patients were first diagnosed from January 2008 to November 2011 at the Second People’s Hospital of Ji’nan. The diagnosis of CAD was based on angiographic evidence of ≥70% stenosis of one major coronary artery and/or≥50% of the left main coronary artery. Initially, a total of 287 patients were collected, and 260 patients agreed to participate in our study, with a participation rate of 90.6%. A total of 313 controls who received a regular health examination were included into control group. Finally, 281 controls were enrolled in our study, with a participation rate of 89.9%. Controls with known CAD or other heart disease were excluded. This study was approved by the ethics committee of the Second People’s Hospital of Ji’nan, and written informed consent was obtained from all the cases and controls. All patients were asked to provide 5 ml venous blood. 

***Genotyping: ***All candidate loci of IL-16 for tag single nucleotide polymorphisms (SNPs) in the NCBI dbSNP database and SNP info. We selected common variants with the minor allele frequency should ≥10% of the Chinese population, and the SNP could influence the microRNA binding sites activity. Finally five SNPs, rs8034928, rs3848180, rs1131445, rs4778889 and rs11556218, were selected.

The genomic DNA was extracted by the method of buffy-coat fractions with TIANamp blood DNA kit (Tiangen Biotech, Beijing, China). Genotyping of rs8034928, rs3848180, rs1131445, rs4778889 and rs11556218 was performed in a 384-well plate format on the Sequenom MassARRAY platform (Sequenom, San Diego, USA), and methods of polymerase chain reaction (PCR) and matrix-assisted laser desorption/ionization time-of-flight (MALDI-TOF) mass spectrometry technologies. The ingle base extension (SBE) and PCR primers were designed using Sequenom Assay Design 3.1 software (Sequenom, San Diego, CA, USA) (Table-I). Each PCR reaction was carried out with a volume of 20 μl containing 50ng of genomic DNA, 200μM dNTP, 2.5 units of Taq DNA polymerase, and 200μM primers. The cycling programme of PCR reaction was preliminary denaturation at 94°C for 2 min, followed by 35 cycles of 94°C for 30 s, annealing temperature reduced to 64°C for 30 s, and 72°C for 10 min. PCR product was testified by on 1.0% agrose gel electrophoresis. A repeat analysis of a randomly chosen subgroup of 10% of the cases and controls was conducted for quality control, and the reproducibility was 100%.

**Table-I:** PCR amplification primers and restriction enzymes

*IL-16 SNP*	*Forward primer*	*Reverse primer*
rs8034928	5´-TTCCATTTGAAGAGAGC-3´	5´-TGCAGAAAACCCAGGTTC-3´
rs3848180	5´-CCTCCAGTTCACAGCATCA-3´	5´-GCTCTACGTTAGTTCCCTTA-3´
rs1131445	5´-TTGATGTTGGCTGGGAACT-3´	5´-CACGCTTTGAGCTTGGTG-3´
rs4778889	5´-CTCCACACTCAAAGCCTTT-3´	5´-CCATGTCAAAACGGTAGCCT-3´
rs11556218	5´-CTCAGGTTCACAGAGTGT-3´	5´-TGTGACAATCACAGCTTGCC-3´

***Statistical analysis:*** Continuous variables were shown as mean±SD and analyzed by students t test. Categorical variables were expressed as frequency and percentage and analyzed by chi-square test. The Hardy-Weinberg equilibrium and genotype distributions between groups were analyzed using chi-square test. Odds ratios (OR) and the corresponding 95% confidence intervals (CI) were taken to evaluate the effect of IL-16 (rs8034928, rs3848180, rs1131445, rs4778889 and rs11556218) on the risk of CAD. Multivariable logistic regression analysis was conducted to calculate the OR (95% CI) after adjusting for potential confounding factors of CAD, such as sex, smoking, BMI, hypertension, diabetes, TC, TG, LDL-C and HDL-C. All statistical analyses were conducted by SPSS 11.0 software (SPSS, Chicago, IL), and *P* value<0.05 was regarded as statistically significant.

## RESULTS

This study included 260 CAD cases (172 males and 88 females, mean age of 63.1±7.8 years, range 42 to 79 years) and 281 health controls (152 males and 129 females, mean age of 62.4±8.5 years, range 40 to 74 years) (Table-II). Compared with the controls, the CAD patients were more likely to be male, had older age, higher BMI and hypertension, and higher proportion of smoking status and diabetes (*P*<0.05 for all comparisons). The control group had significantly higher TC, HDL-C and LDL-C levels when compared with CAD patients.

**Table-II:** The clinical characteristics between CAD patients and controls

*Variables*	*Cases, n*	*%*	*Controls, n*	*%*	*P value*
Age (years)	63.1±7.8		62.4±8.5		0.1
Sex (%)					
Male	172	66.2	152	54.2	<0.05
Female	88	33.8	129	45.8	
BMI (kg/m^2^)		23.4±3.2		22.5±2.6	<0.05
Smoking (%)					
Ever	103	39.5	86	30.6	
Never	157	60.5	195	69.4	<0.05
Diabetes (%)					
Yes	108	41.7	55	19.7	
No	152	58.3	226	80.3	<0.05
Hypertension (%)					
Yes	85	32.8	60	21.2	
No	175	67.2	221	78.8	<0.05
TC(mmol/L)	4.1±1.2		4.6±1.3		<0.05
TG(mmol/L)	2.0±1.2		1.7±1.0		<0.05
LDL-C(mmol/L)	2.6±1.1		2.9±0.8		<0.05
HDL-C(mmol/L)	1.2±0.4		1.5±0.4		<0.05

**Fig.1:**
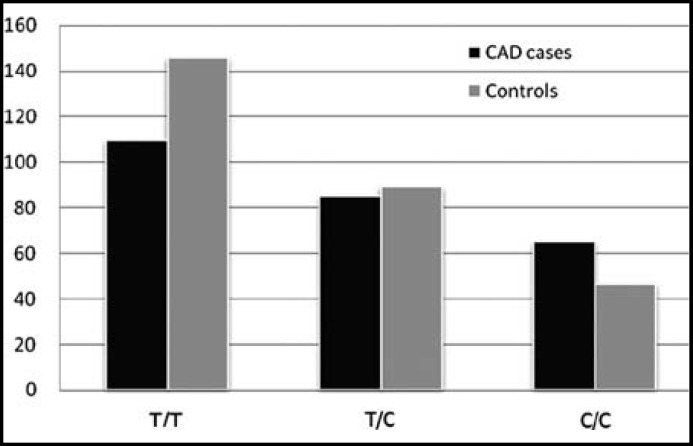
Genotype frequencies of rs8034928

**Fig.2:**
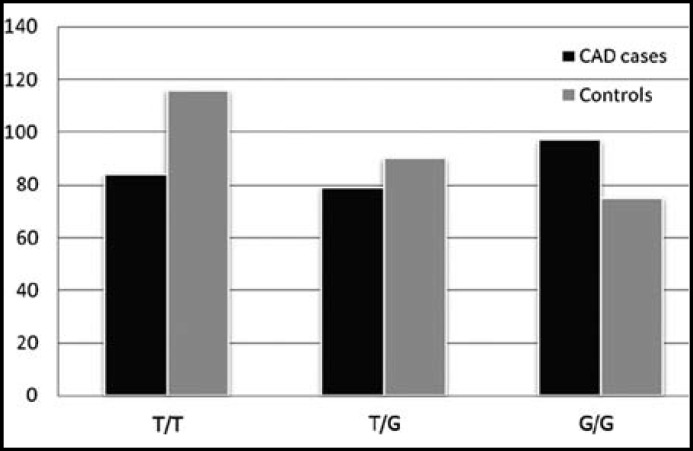
Genotype frequencies of rs3848180

The genotype distributions of the five SNPs were shown in Table-III. The minor allele frequencies of IL-16 rs8034928, rs3848180, rs1131445, rs4778889 and rs11556218 in controls were in line with the published MAFs (http://www.ncbi.nlm.nih.gov/snp/) and the Hardy-Weinberg equilibrium. The IL-16 rs8034928 and rs3848180 genotype frequencies showed significantly difference between the two groups, and the IL-16 rs8034928 C allele and rs3848180 G allele frequencies were significantly higher in CAD patients than controls (57.7% vs 48.1% for rs8034928 C allele; 67.7% vs 58.7% for rs3848180 G allele) (Fig. 1 and 2). However, there was no significant difference between the genotype frequencies of IL-16 rs1131445, rs4778889 and rs11556218 (*P*>0.05).

**Table-III:** Distribution of IL-16 genes polymorphisms between CAD cases and controls

*IL-16 SNP*	*Major/Minor allele*	*MAF in PubMed*	*Minor frequencies*		*CAD cases, n*			*Controls, n*			*P value*
			*Case*	*Control*	*A/A* ^1^	*A/a* ^2^	*a/a* ^3^	*A/A*	*A/a*	*a/a*	
rs8034928	T/C	0.327	0.414	0.324	110	85	65	146	89	46	<0.05
rs3848180	T/G	0.477	0.446	0.428	84	79	97	116	90	75	<0.05
rs1131445	T/C	0.346	0.367	0.346	120	90	50	137	95	50	0.83
rs4778889	C/T	0.315	0.297	0.301	139	89	33	151	91	39	0.87
rs11556218	G/T	0.211	0.204	0.210	175	64	21	191	63	28	0.67

1. Wild-type; 2. Heterozygous variant; 3. Homozygous variant

Multivariate logistic regression analysis was taken to analyze the effect of IL-16 rs8034928 and rs3848180 polymorphisms on the CAD risk, adjusted for sex, BMI, smoking, diabetes, hypertension, TC, HDL-C, LDL-C and TG. The IL-16 rs8034928 C/C genotype showed a significant increased risk of CAD in codominant mode (OR=1.87, 95%CI=1.17-3.03), and the variant of IL-16 rs8034928 showed a significant increased risk of CAD in dominant (OR=1.48, 95%CI=1.04-2.10) and recessive model (OR=1.70, 95%CI=1.10-2.67). For polymorphism of IL-16 rs3848180, the IL-16 rs3848180 G/G genotype showed increased risk of CAD after adjusting potential risk factors (OR=1.79, 95%CI=1.16-2.75), and G allele genotype was associated with increased CAD risk in dominant model (OR=1.47, 95%CI=1.02-2.13).

**Table-IV:** Association between polymorphisms in IL-6 and IL-16 and CAD risk

*IL-16 SNP*		*Minor allele frequencies*				*OR (95% CI)* ^1^		
		*Cases*	*%*	*Controls*	*%*	*Codominant*	*Dominant*	*Recessive*
rs8034928	T/T	110	42.3	146	52.0	-	-	-
	T/C	85	32.7	89	31.7	1.27(0.85-1.90)	1.48(1.04-2.10)	1.70(1.10-2.67)
	C/C	65	25.0	46	16.4	1.87(1.17-3.03)		
rs3848180	T/T	84	32.3	116	41.3	-		
	T/G	79	30.4	90	32.0	1.21(0.79-1.87)	1.47(1.02-2.13)	1.09(0.76-1.57)
	G/G	97	37.3	75	26.7	1.79(1.16-2.75)		

1. Adjusted for sex, BMI, smoking, diabetes, hypertension, TC, HDL-C, LDL-C and TG

The combined effect of IL-16 rs8034928 and rs3848180 on the CAD risk was analyzed by multivariate logistic regression analysis, adjusted for sex, BMI, smoking, diabetes, hypertension, TC, HDL-C, LDL-C and TG. Individuals carrying both IL-16 rs8034928 C allele and rs3848180 T/T genotypes significantly increased the risk of CAD when compared with those carrying both IL-16 rs8034928 T/T and rs3848180 T/T genotypes (OR=2.31, 95% CI=1.24-4.32). Moreover, individuals with both IL-16 rs8034928 T/T and rs3848180 G allele genotypes had a significant increased risk of CAD than double wild-type genotypes (OR=2.02, 95% CI=1.18-3.45).

**Table-V:** Combined effect of rs8034928 and rs3848180 on the CAD risk

*IL-16 SNP*		*Cases*	*%*	*Controls*	*%*	*OR (95% CI)* ^1^
*rs8034928*	*rs3848180*					
T/T	T/T	42	16.2	81	28.8	-
C allele	T/T	42	16.2	35	12.5	2.31(1.24-4.32)
T/T	G allele	68	26.2	65	23.1	2.02(1.18-3.45)
C allele	G allele	108	41.4	126	35.6	1.56(0.93-2.67)

1. Adjusted for sex, BMI, smoking, diabetes, hypertension, TC, HDL-C, LDL-C and TG

## DISCUSSION

In the present study, we have found that variants of IL-16 rs8034928 and rs3848180 may be associated with CAD risk in a Chinese population. Our study suggests that IL-16 rs8034928 and rs3848180 polymorphisms may influence the susceptibility to CAD risk. 

Recently, the identification of novel genetic variants for evaluating the early risk of CAD is attracting increasing interest of investigators on cancer risk worldwide. Based on the genetic information, we could determine the disease etiology in terms of genetic determinants to be used for identifying the high-risk individuals and perform targeting therapy to the individual’s genetic make-up.^11^

It is reported that IL-16 gene consists of 7 exons and 6 introns convering approximately 12.8kb of genomic DNA.^12^ The polymorphisms of IL-16 rs8034928 and rs3848180 polymorphisms are located on the intron region, and thus the polymorphisms could influence the susceptibility of human disease. Previous studies have reported that polymorphism of IL-16 rs11556218 and rs8034928 T/C are associated with various human cancers, such as allergic contact dermatitis, systemic lupus erythematosus, nasopharyngeal carcinoma and renal cell carcinoma.^6^^-^^8^^,^^13^ Previous two studies have shown that polymorphisms of IL-16 rs11556218 and rs8034928 are associated with risk of CAD.^14^^,^^15^

A study conducted in China with 651 CAD patients and 428 health controls has showed that rs8034928 C/C genotype is associated with a significant increased risk of CAD in a Chinese population, with the OR(95% CI) of 1.33(1.15-1.47).^14^ In additionally, this study has shown that haploptypes TTTT and TGGT of IL-16 rs8034928, rs3848180 and rs1131445 significantly increased risk to CAD, whereas haplotypes of CTTT and TTGT referred to protection of CAD.^14^ Similarly, we have found that IL-16 rs8034928 polymorphism is associated with a higher risk of CAD in our study, which is in line with previous reports.^14^^,^^15^

A recent study has shown that intronic sequences encode microRNAs, which are associated with RNA-mediated gene silencing.^16^ This study has shown that IL-16 rs8034928 T/C polymorphism is in linkage disequilibrium with other casual genetic variants.^16^ In our study, we found that IL-16 rs8034928 T/C polymorphism could influence the risk of CAD. Huang has reported that IL-16 rs3848180 polymorphism is associated with risk of CAD, and the rs3848180 polymorphism may influence IL-16 rs8034928 gene expression.^14^ The results of our study also support the association between polymorphism of IL-16 rs8034928 and CAD risk. Further confirmation of our results is strongly needed in future studies.

There are several limitations in our study. Firstly, the subjects were collected in a single hospital, and the samples could not well represent all the other populations. Secondly, CAD is a disease induced by multiple genes and environmental factors, and more genetic and environmental factors should be considered in further study.

In conclusion, our study indicates that the variants of IL-16 rs8034928 and rs3848180 are associated with risk of CAD. However, we did not find statistically association of polymorphisms in rs1131445, rs4778889 and rs11556218 of IL-16 gene with CAD risk. The IL-16 gene polymorphisms may be used as a predictor to the susceptibility of CAD.

## Authors Contributions:

**THF & WW** designed and performed the study, did statistical analysis & editing of manuscript.

**YYY, ZJ, GSP & LHM** did data collection and manuscript writing.
